# Mega-electron-volt Microcrystal Electron Diffraction

**DOI:** 10.1063/4.0000962

**Published:** 2025-10-27

**Authors:** Xiaozhe Shen, Duan Luo, Xijie Wang

**Affiliations:** 1 Institute of Advanced Science Facilities, Shenzhen, Guangdong, China; 2 Key Laboratory of Ultrafast Photoelectric Diagnostics Technology, Xi’an Institute of Optics and Precision Mechanics, Chinese Academy of Sciences, Xi’an, Shaanxi, China; 3 University of Duisburg-Essen, Duisburg, North Rhine-Westphalia, Germany; 4 TU Dortmund University, Dortmund, North Rhine-Westphalia, Germany

## Abstract

Characterization of atomic structure in matter benefits significantly from mega-electron-volt (MeV) electron beams, which offer enhanced penetration depths and substantially reduced multiple scattering effects compared to conventional kilo-electron-volt (keV) electron beams. MeV ultrafast electron diffraction (UED) has demonstrated broad and transformative impacts in ultrafast science, covering the field of condensed matter physics [1-4], and gas-phase [5-6] as well as liquid-phase chemistry [7-8]. Recent advancements in generation of micro- focused MeV beam [9] and the development of single-MeV-electron-sensitive detector [10] have paved the way for a novel application frontier: MeV microcrystal electron diffraction (microED). In this presentation, we report the first demonstration of MeV microED to atomic-scale structure characterization of a two-dimensional molecular crystal (2DMC) called C6-DPA. 2DMCs have emerged as a novel platform to explore structure–property relationships for the design of high-performance optoelectronic devices using small molecules. As a promising 2DMC material, the crystal structure of C6-DPA remains incompletely resolved. To address this, we applied MeV microED to acquire a full tilt series of diffraction patterns for structure refinement of C6-DPA. Our results achieved a spatial resolution better than 1 Å (see Fig. 1) under low-dose condition, with a dose rate of 5.2E−7 e-/Å^2^/s and a total dose of 1.1E−2 e-/Å^2^. The experimental setup, data acquisition protocols and structure retrieval analysis will be discussed in details. Finally, we will outline future technical developments to further enhance capabilities of MeV microED for atomic-resolution characterization of biological materials.
